# Prenatal determinants of physical activity and cardiorespiratory fitness in adolescence – Northern Finland Birth Cohort 1986 study

**DOI:** 10.1186/s12889-017-4237-4

**Published:** 2017-04-20

**Authors:** Marjaana Tikanmäki, Tuija Tammelin, Marja Vääräsmäki, Marika Sipola-Leppänen, Satu Miettola, Anneli Pouta, Marjo-Riitta Järvelin, Eero Kajantie

**Affiliations:** 1 Chronic Disease Prevention Unit, Department of Health, National Institute for Health and Welfare, Oulu and Helsinki, Finland; 2 Institute of Health Sciences, University of Oulu, Oulu, Finland; 3 LIKES Research Center for Physical Activity and Health, Jyväskylä, Finland; 4 Pediatrics and Adolescence and Obstetrics and Gynecology, Medical Research Center Oulu, Oulu University Hospital and University of Oulu, Oulu, Finland; 5 Children, Adolescents and Families Unit, Department of Welfare, National Institute for Health and Welfare, Oulu, Finland; 6 Department of Government Services, National Institute for Health and Welfare, Helsinki, Finland; 7 Department of Epidemiology and Biostatistics, MRC–PHE Centre for Environment & Health, School of Public Health, Imperial College London, London, UK; 8 Center for Life Course Epidemiology, Faculty of Medicine, University of Oulu, Oulu, Finland; 9 Biocenter Oulu, Oulu, Finland; 10 Unit of Primary Care, Oulu University Hospital, Oulu, Finland; 11 Children’s Hospital, Helsinki University Hospital, University of Helsinki, Helsinki, Finland

**Keywords:** Exercise, Birth weight, Length of gestation, Preterm birth, Gestational diabetes, Gestational hypertension, Obesity, Overweight, Smoking during pregnancy, Cardiometabolic risk factors

## Abstract

**Background:**

Lower levels of physical activity and cardiorespiratory fitness are key risk factors of chronic adult diseases. Physical activity and cardiorespiratory fitness are predicted by birth weight, but the underlying parental and pregnancy-related factors remain largely unknown. We examined how prenatal determinants are associated with physical activity and cardiorespiratory fitness in adolescence.

**Methods:**

Of the 16-year-old members of the population-based Northern Finland Birth Cohort 1986 (NFBC 1986), 6682 singletons with no major physical disability reported their amount of physical activity outside school hours, and 4706 completed a submaximal cycle ergometer test assessing cardiorespiratory fitness. Physical activity was expressed as metabolic equivalent hours per week (METh/week) and cardiorespiratory fitness as peak oxygen uptake (ml·kg^−1^·min^−1^). Prenatal determinants included birth weight, length of gestation, mother’s and father’s body mass index (BMI), maternal gestational diabetes mellitus (GDM), and maternal hypertension and smoking during pregnancy. Data were analyzed by multiple linear regression.

**Results:**

A higher birth weight and longer length of gestation predicted lower levels of physical activity and cardiorespiratory fitness at 16 years, although the association between length of gestation and physical activity was inverse U-shaped. Mother’s or father’s overweight or obesity before pregnancy were associated with lower levels of their offspring’s physical activity and fitness in adolescence. Adjusting for maternal pregnancy disorders and the adolescent’s own BMI attenuated the associations with the mother’s but not the father’s overweight/obesity. Furthermore, maternal GDM predicted lower cardiorespiratory fitness.

**Conclusions:**

A high birth weight and parental overweight/obesity are associated with lower levels of both physical activity and cardiorespiratory fitness in adolescence, while maternal GDM and longer length of gestation are associated with lower cardiorespiratory fitness. Both long and short lengths of gestation predict low physical activity.

**Electronic supplementary material:**

The online version of this article (doi:10.1186/s12889-017-4237-4) contains supplementary material, which is available to authorized users.

## Background

Low levels of physical activity and cardiorespiratory fitness are important risk factors of cardiometabolic and other non-communicable disease and all-cause mortality [1–4]. It has recently become increasingly clear that much of the risk of these diseases originates from fetal life, and markers of fetal life conditions, such as low birth weight [5–10], preterm birth [11], hypertension in pregnancy [12–16], high maternal BMI [17], gestational diabetes [18] and maternal smoking [19–21] have been linked with adult hypertension, coronary heart disease, stroke, type 2 diabetes or their premorbid risk factors. The mechanisms of this fetal “programming” remain poorly known, and whether programming of physical activity and fitness act as mediating factors is not known. To elucidate this, the first step would be to assess whether fetal life markers predict these outcomes. Existing studies have, however, largely been limited to birth weight and preterm birth as markers.

A meta-analysis of 13 questionnaire-based studies suggested that both low and high extremes of birth weight were associated with a lower level of leisure-time physical activity in adolescence and adulthood [22]. The low extreme likely reflects mostly preterm births at very (<1500 g) or extremely (<1000 g) low birth weights, which are associated with lower self-reported levels of physical activity [23–25] and, in most studies, lower cardiorespiratory fitness [25–30]. Few studies have assessed these outcomes in people with high birth weights [31, 32].

With this background, we examined the associations of birth weight and underlying prenatal and parental characteristics with physical activity and cardiorespiratory fitness in adolescence among the members of a birth cohort.

## Methods

### Study cohort

The Northern Finland Birth Cohort 1986 (NFBC 1986) is a longitudinal birth cohort comprising all births with an expected date of delivery between July 1, 1985 and June 30, 1986 in the two northernmost provinces of Finland, involving 9479 cohort members [33, 34].

At age 15–16 years, all traceable cohort members (*N* = 9215) received a postal questionnaire including questions on physical activity and were invited to participate in a clinical examination which included measurement of cardiorespiratory fitness. Altogether, 7344 (80%) responded to the questionnaire, and 6798 (74%) attended the examination [35]. The parents were also sent a questionnaire that included questions on socioeconomic position and lifestyle to which 6985 (76%) responded (Additional file 1).

The present study includes individuals with no major neurosensory impairment, who had appropriate data on self-reported physical activity (6682; 3189 boys and 3493 girls) or on cardiorespiratory fitness (4706; 2513 and 2193) (Additional file 1).

### Prenatal determinants

#### Birth weight and length of gestation

Prenatal and neonatal data were extracted from hospital records. *Length of gestation* was determined as described [36]. *Birth weights* were converted into standard-deviation scores relative to sex and length of gestation according to Finnish birth weight charts [37]. Frequencies in categories of birth weight, length of gestation, and birth weight SD are shown in Additional file 2.

### Maternal pregnancy conditions

To screen for *maternal hypertensive disorders during pregnancy*, blood pressure and a urinary protein dip-stick test were performed during every visit at the maternity welfare clinic. Blood pressure > 140/90 mmHg or the use of antihypertensive medication before 20 weeks of gestation were considered chronic hypertension. Gestational hypertension was defined as blood pressure ≥ 140/90 mmHg after 20 weeks of gestation. A positive urinary dip-stick test (≥ 0.3 g/L) indicated proteinuria in a normotensive (blood pressure < 140/90 mmHg) mother, preeclampsia in a mother with blood pressure ≥ 140/90 mmHg, and superimposed preeclampsia in a mother with chronic hypertension [38].

*Maternal gestational diabetes mellitus (GDM)* was screened by oral glucose tolerance test (OGTT) performed mainly at 26 to 28 gestational weeks, according to prevailing national guidelines [34], if any of the following risk factors were present: glucosuria, prior GDM, suspected fetal macrosomia, previous macrosomic infant (birth weight > 4500 g), maternal body mass index, BMI >25, and maternal age > 40 years. GDM was diagnosed if the OGTT yielded one or more abnormal values of capillary blood glucose concentration (exceeding 5.5, 11.0, or 8.0 mmol/L at fasting, one hour and two hours, respectively). The reference group consisted of subjects whose mothers had no GDM risk factors and thus did not undergo OGTT. The three exposure groups included: 1) offspring exposed to maternal GDM; 2) offspring of normal weight mothers (BMI < 25) with normal OGTT; and 3) offspring of overweight or obese mothers with normal OGTT.

*Maternal smoking during pregnancy.* “Did you smoke after the second month of pregnancy? Yes/No” was asked by questionnaire.

### Parental BMI

*Maternal BMI before pregnancy* (kg/m2) and *father’s BMI at the beginning of pregnancy* were calculated based on weight and height, self-reported at the beginning of pregnancy.

### Outcomes

#### Self-reported physical activity at 16 years

The amount of physical activity outside school hours at 16 years was evaluated by asking “How many hours a week altogether do you participate in (a) brisk and (b) light physical activity outside school hours?” (response alternatives: not at all, about ½ hour, about 1 h, 2–3 h, about 4–6 h, 7 h or more a week) and “How many minutes altogether does it take you to walk, cycle or otherwise physically move to school and back home daily? ” (response alternatives: not at all, less than 20 min, 20–39 min, 40–59 min, at least 1 h per day) [39] (Additional file 3). In the questionnaire, the term “brisk” was described as physical activity causing at least some sweating and getting out of breath (here referred to as moderate-to-vigorous physical activity, MVPA; assuming a value of 5 METs), and the term “light” as physical activity causing no sweating or shortage of breath (light physical activity, LPA; 3 METs) and commuting physical activity walking or cycling (CPA, 4 METs). Overall physical activity level was estimated using information on MVPA, LPA, and CPA by calculating physical activity levels in metabolic equivalent hours (METh) per week as described [39, 40]. The test-retest reliability of these questions is fairly good among Finnish 15- to 16-year-olds [39].

### Cardiorespiratory fitness at 16 years

In conjunction with the clinical examination, cardiorespiratory fitness was measured by a submaximal cycle ergometer test using a two-stage exercise protocol designed to fit examining maximal workload and peak oxygen consumption in the clinical examination of a cohort study. Peak oxygen uptake was calculated based on heart rate responses during two submaximal work stages and expressed as ml·kg^−1^·min^−1^. The protocol has been described and validated against the maximal cycle ergometry test [35].

### Covariates

*Sex of the participant* and an indicator of socio-economic position, *the educational attainment of the higher educated parent* (four categories) at the offspring age 16, were taken into account as covariates.

The covariates further included *parental and pregnancy-related factors* (gestational age, maternal GDM, maternal hypertension, maternal and paternal BMI, maternal smoking).

*Physical activity of the mother and father* at the offspring age 16, “How often do you participate in brisk physical activities/ exercise during your leisure time?” with five response options [41] was used as a general lifestyle indicator of the family over time [42].

*BMI* at 16 years was calculated (kg/m^2^) primarily according to the measurements at the clinical examination. For 762 (11.4%) individuals in physical activity analyses, only self-reported height and weight were available because they did not attend the clinical visit. *Pubertal stage* at 16 years was inquired by a structured questionnaire based on drawings illustrating different Tanner stages [43]. *Smoking at 16 years* was categorized to smokers (defined as regular smoking in the past or current smoking weekly or more frequently) and others (non-smokers, those smoking less than weekly, and those with no information). *Season of the year* during study was defined for both outcomes: spring (March–May), summer (June–August), autumn (September–November), and winter (December–February).

### Statistical methods

We used multiple linear regression for continuous outcome variables. We tested for non-linear relationships by including a quadratic term in the model and for interactions between two variables by including a product term together with these variables. All analyses were performed using IBM SPSS Statistics, Version 21.

Continuous explanatory variables were first tested for linear and quadratic association as such and then categorized and entered as dummy variables, with one category treated as reference. Categorical variables were entered as dummy variables, with a separate dummy variable indicating missing values. In Model 1, the results were adjusted for sex. Model 2 was in addition adjusted for educational attainment of the higher educated parent. We also introduced three parallel models. Model 3a included sex, parental education, and parental and pregnancy-related factors (gestational age, maternal GDM, maternal hypertension, maternal BMI before pregnancy, paternal BMI at the beginning of pregnancy, maternal smoking). Model 3b included sex, parental education, and physical activity of parents as an indicator of the lifestyle of the family over time. Model 3c included sex, parental education, and the adolescent’s current characteristics (the adolescent’s BMI, age, pubertal stage, smoking), and season.

The analysis was rerun by including only participants with no missing data (complete case analysis). Furthermore, Models 1 and 2 were rerun after the exclusion of participants with asthma. Characteristics of participants and non-participants were compared with χ^2^-tests for categorical and Student’s t-test for continuous variables.

## Results

The characteristics of the participants and non-participants are presented for physical activity study in Additional file 4 and for cardiorespiratory fitness study in Additional file 5.

### Physical activity and cardiorespiratory fitness

The mean level of self-reported physical activity at 16 years was 30.7 (SD 16.8) METh/week for all and higher for boys compared to girls (33.0 [SD 17.9] vs 28.7 [SD 15.5] METh/week) (*p* < 0.001). The mean estimate of peak oxygen uptake, an indicator of cardiorespiratory fitness, was 42.7 (SD 10.7) ml·kg^−1^·min^−1^ for all and, higher in boys than girls (49.1 [SD 9.7] vs 35.4 [SD 6.3] ml·kg^−1^·min^−^1) (*p* < 0.001). Mean values of the outcomes in exposure categories are presented in Additional file 6. The sex-adjusted correlation coefficient between physical activity and cardiorespiratory fitness was 0.22. Associations between covariates and outcomes are shown in Additional file 6.

### Birth weight and birth weight SD score

**Fig. 1:**
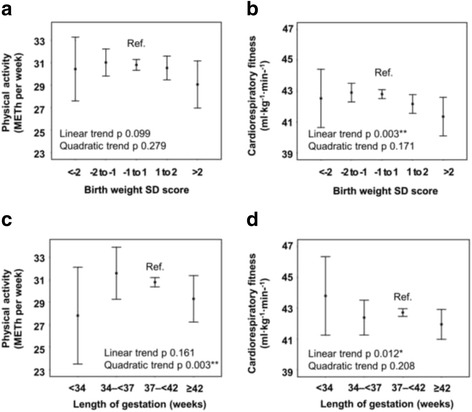
Birth weight SD score and length of gestation predicting physical activity and cardiorespiratory fitness. Sex adjusted mean levels (95% CI) of physical activity (METh/week) and cardiorespiratory fitness (ml·kg^−1^·min^−1^) in adolescents according to exposure. *P* values are for linear and quadratic trends for continuous variables. No statistically significant mean differences between categories were found compared with the reference category (Ref.)

A one kilogram higher birth weight was associated with an 0.6 METh/week (95% CI -0.2 to 1.4, adjusted for sex) lower level of physical activity and an 0.8 ml·kg^−1^·min^−1^ (0.3 to 1.3) lower cardiorespiratory fitness. There were no quadratic trends (*p* > 0.06). Similar linear associations were observed with birth weight SD score as a predictor. (Table 1 and Fig. 1).

**Table 1:** Birth weight SD score in association with physical activity and cardiorespiratory fitness at 16 years. Mean differences (95% CI) of physical activity (METh/week) and cardiorespiratory fitness (ml·kg^−1^·min^−1^) per one unit higher birth weight SD score

	Model	Mean difference per one unit higher value (95% CI)
Physical activity (METh/week)		*N* = 6675
	1	−0.3 (−0.7;0.1)
	2	−0.4 (−0.8;0.0)*
	3a	−0.3 (−0.7;0.1)
	3b	−0.4 (−0.8;0.0)*
	3c	−0.5 (−0.9;-0.1)*
	1	*P* for quadratic trend 0.279
Cardiorespiratory fitness (ml·kg^−1^·min^−1^)		*N* = 4701
	1	−0.4 (−0.6;-0.1)**
	2	−0.4 (−0.6;-0.1)**
	3a	−0.3 (−0.6;0.0)*
	3b	−0.4 (−0.6;−0.1)*
	3c	-0.1 (−0.3;0.1)
	1	*P* for quadratic trend 0.171

Multiple linear regression models are adjusted for:

Model 1: sex

Model 2: sex, parental education

Model 3a: Model 2 + length of gestation, maternal GDM, maternal hypertension, BMI of the mother before pregnancy, BMI of the father at the beginning of pregnancy, smoking of the mother during pregnancy

Model 3b: Model 2 + physical activity of mother and father at the offspring age 16

Model 3c: Model 2 + adolescent’s BMI, age, pubertal stage, and smoking, and season of the year at the time of the study

*P* values are for mean difference between risk factor groups and controls: * < 0.05, ** < 0.01, and quadratic trend. Categorical covariates are dummy-coded, with a separate dummy variable for missing values

### Length of gestation

There was an inverse U-shaped association between length of gestation and physical activity such that adolescents born at both ends of the full range of the length of gestation undertook less physical activity than others (Table 2, Fig. 1). These adolescents also seemed to have lower cardiorespiratory fitness; however, only the linear inverse trend was statistically significant (Table 2, Fig. 1), suggesting lower fitness in those born with longer lengths of gestation.

**Table 2:** Length of gestation (weeks) in association with physical activity and cardiorespiratory fitness at 16 years. Mean differences (95% CI) of physical activity (METh/week) and cardiorespiratory fitness (ml·kg^−1^·min^−1^) per one unit higher value

	Model	Mean difference per one unit higher value (95% CI)
Physical activity (METh per week)		*N* = 6675
	1	−0.2 (−0.4;0.1)
	2	−0.2 (−0.4;0.1)
	3a	−0.2 (−0.5;0.1)
	3b	−0.2 (−0.4;0.1)
	3c	−0.2 (−0.4;0.1)
	1	*P* for quadratic trend 0.003*
Cardiorespiratory fitness (ml·kg^−1^·min^−1^)		*N* = 4701
	1	−0.2 (−0.3;0.0)*
	2	−0.2 (−0.3;0.0)*
	3a	−0.2 (−0.4;-0.0)*
	3b	−0.2 (−0.3;0.0)*
	3c	−0.1 (−0.3;0.0)
	1	*P* for quadratic trend 0.208

Multiple linear regression models are adjusted for:

Model 1: sex

Model 2: sex, parental education

Model 3a: Model 2 + birth weight SD score, maternal GDM, maternal hypertension, BMI of the mother before pregnancy, BMI of the father at the beginning of pregnancy, smoking of the mother during pregnancy

Model 3b: Model 2 + physical activity of mother and father at the offspring age 16

Model 3c: Model 2 + adolescent’s BMI, age, pubertal stage and smoking, and season of the year at the time of the study

*P* values are for mean difference between risk factor groups and controls: * < 0.05, and quadratic trend. Categorical covariates are dummy-coded, with a separate dummy variable for missing values

### Maternal BMI

**Fig. 2:**
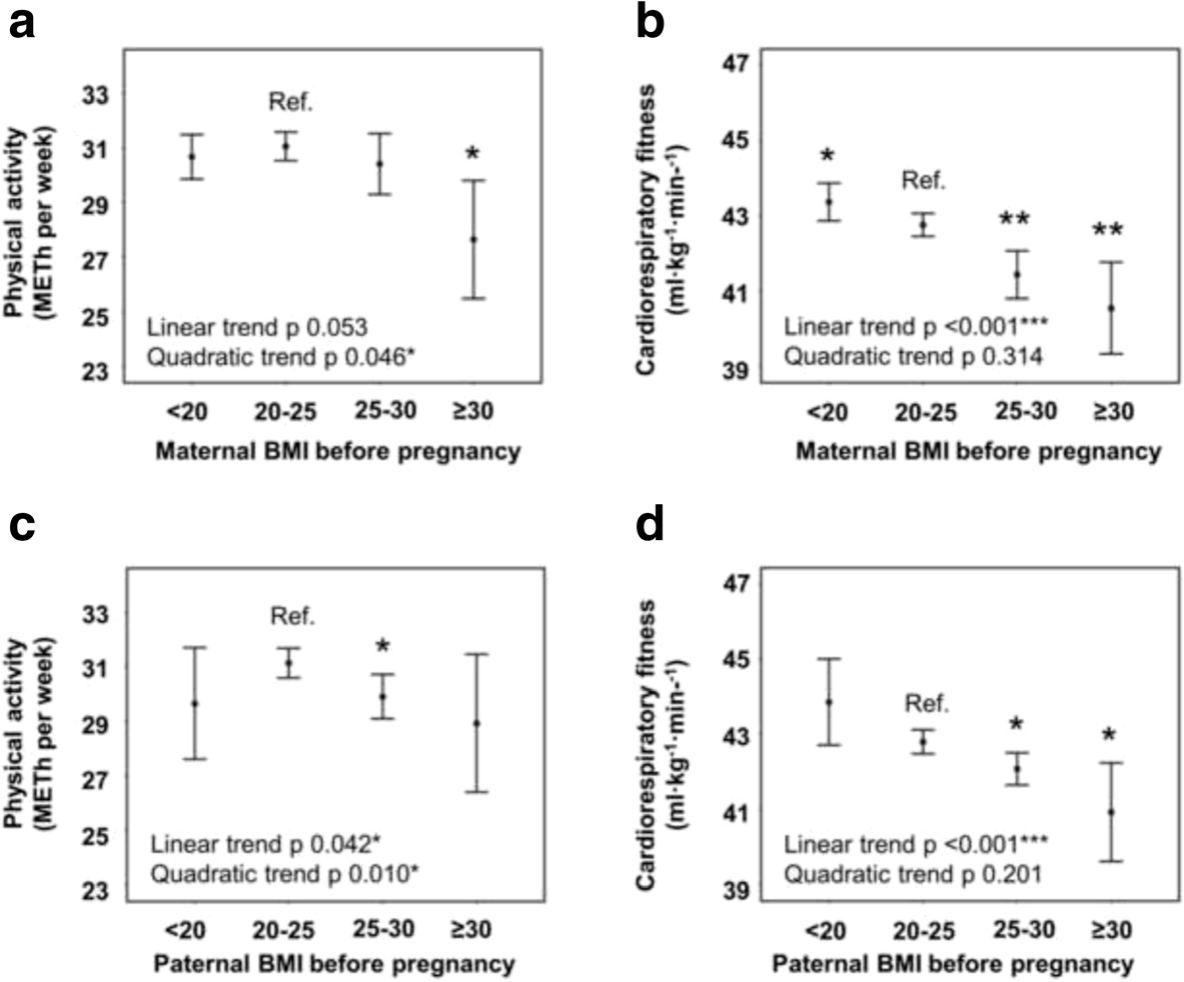
Maternal and paternal BMI before pregnancy predicting physical activity and cardiorespiratory fitness. Sex adjusted mean levels (95% CI) of physical activity (METh/week) and cardiorespiratory fitness (ml·kg^−1^·min^−1^) in adolescents according to exposure. *P* values are for linear and quadratic trends for continuous variables, and statistically significant mean differences compared with the reference category (Ref.) are shown as *p* value * < 0.05, ** < 0.01, *** < 0.001

Adolescents whose mothers were obese before pregnancy undertook less physical activity than those of normal weight mothers (Table 3, Fig. 2). There was also an inverse association between maternal pre-pregnancy BMI and adolescents’ cardiorespiratory fitness (−0.2 ml·kg^−1^·min^−1^ for each unit BMI, 95% CI -0.3 to −0.2). The association was statistically significant in both sexes, but it was stronger in boys (p for interaction 0.03).

**Table 3:** Maternal BMI before pregnancy in association with physical activity and fitness among adolescents. Mean differences (95% CI) of physical activity (METh/week) and cardiorespiratory fitness (ml·kg^−1^·min^−1^) in adolescents whose mothers were underweight, overweight, or obese before pregnancy compared with offspring of normal weight mothers

	Model	BMI category (kg/m^2^)			
		< 20	20–25	25–30	> 30
		Underweight	Normal weight	Overweight	Obese
Physical activity (METh/week)		*N* = 1594	*N* = 3881	*N* = 829	*N* = 229
	1	−0.4 (−1.4;0.6)	Mean 31.0 (SD 16.9)	−0.6 (−1.9;0.6)	−3.4 (−5.6;-1.2)*
	2	−0.3 (−1.3;0.6)		−0.3 (−1.5;1.0)	−2.7 (−5.0;−0.5)*
	3a	-0.5 (−1.5;0.5)		0.7 (−0.7;2.2)	−1.6 (−4.0;0.7)
	3b	−0.2 (−1.2;0.7)		−0.0 (−1.3;1.2)	−2.2 (−4.4;0.0)
	3c	−0.1 (−1.1;1.0)		−0.2 (−1.5;1.0)	−2.3 (−4.5;0.0)*
Cardiorespiratory fitness (ml·kg^−1^·min^−1^)		*N* = 1122	*N* = 2760	*N* = 576	*N* = 148
	1	0.6 (0.0;1.2)*	Mean 42.6 (SD 10.7)	−1.3 (−2.1;-0.6)**	−2.2 (−3.6;-0.9)**
	2	0.6 (0.1;1.2)*		−1.2 (−2.0;-0.5)**	−2.0 (−3.4;-0.6**
	3a	0.5 (−0.1;1.1)		−0.8 (−1.7;0.1)	−1.5 (−2.9;0.0)
	3b	0.6 (0.1;1.2)*		−1.2 (−1.9;-0.4)**	−1.9 (−3.3;−0.5)**
	3c	0.0 (−0.5;0.5)		-0.5 (−1.3;0.2)	0.3 (−1.0;1.6)

Multiple linear regression models are adjusted for:

Model 1: sex

Model 2: sex, parental education

Model 3a: Model 2 + length of gestation, maternal GDM, maternal hypertension, BMI of the father at the beginning of pregnancy, smoking of the mother during pregnancy

Model 3b: Model 2 + physical activity of mother and father at the offspring age 16

Model 3c: Model 2 + subject’s BMI, age, pubertal stage and smoking, and season of the year at study

*P* values are for mean difference between risk factor groups and controls: * < 0.05, ** < 0.01. Categorical covariates are dummy-coded, with a separate dummy variable for missing values

### Paternal BMI

Adolescents whose fathers were overweight at the beginning of pregnancy undertook less physical activity than adolescents of normal weight fathers (Table 4, Fig. 2). With father’s BMI as a continuous variable, there was an inverse linear association with physical activity (−0.2 METh/week for each BMI unit, 95% CI -0.3 to 0.0, adjusted for sex); also an inverse U-shaped quadratic trend was statistically significant (*p* = 0.01). Similarly, lower cardiorespiratory fitness was detected in adolescents whose fathers were overweight or obese (Table 4, Fig. 2). Cardiorespiratory fitness was inversely associated with father’s BMI as a continuous variable (−0.2 ml·kg^−1^·min^−1^, −0.3 to −0.1). This association was statistically significant in both sexes but stronger in boys (*p* for interaction 0.03).

**Table 4:** Paternal BMI before pregnancy in association with physical activity and fitness among adolescents. Mean differences (95% CI) of physical activity (METh/week) and cardiorespiratory fitness (ml·kg^−1^·min^−1^) in adolescents whose fathers were underweight, overweight, or obese at the beginning of the pregnancy compared with offspring of normal weight fathers

	Model	BMI category (kg/m^2^)			
		< 20	20–25	25–30	> 30
		Underweight	Normal weight	Overweight	Obese
Physical activity (METh/week)		*N* = 235	*N* = 3600	*N* = 1619	*N* = 163
	1	−1.5 (−3.7;0.7)	Mean 31.1 (SD 16.8)	−1.2 (−2.2;-0.2)*	−2.2 (−4.8;0.4)
	2	−1.4 (−3.6;0.7)		−1.0 (−2.0;-0.1)*	−1.9 (−4.5;0.7)
	3a	−1.4 (−3.6;0.8)		−1.0 (−2.0;0.0)*	−1.9 (−4.5;0.7)
	3b	−1.5 (−3.6;0.7)		−1.0 (−1.9;0.0)	−1.5 (−4.0;1.1)
	3c	−1.1 (−3.3;1.1)		−1.1 (−2.0;-0.1)*	−1.5 (−4.1;1.1)
Cardiorespiratory fitness (ml·kg^−1^·min-^1^)		*N* = 165	*N* = 2517	*N* = 1141	*N* = 108
	1	1.1 (−0.2;2.3)	Mean 42.8 (SD 10.7)	−0.7 (−1.3;-0.2)*	−1.9 (−3.4;-0.4)*
	2	1.1 (−0.2;2.3)		−0.7 (−1.2;-0.1)*	−1.8 (−3.3;-0.3)*
	3a	1.0 (−0.3;2.3)		−0.6 (−1.1;0.0)	−1.5 (−3.0;0.0)
	3b	1.1 (−0.2;2.3)		−0.6 (−1.2;-0.1)*	−1.7 (−3.2;-0.15)*
	3c	0.6 (−0.6;1.8)		0.0 (−0.5;0.5)	−0.4 (−1.8;1.1)

Multiple linear regression models are adjusted for:

Model 1: sex

Model 2: sex, parental education

Model 3a: Model 2 + length of gestation, maternal GDM, maternal hypertension, BMI of the mother at the beginning of pregnancy, smoking of the mother during pregnancy

Model 3b: Model 2 + physical activity of mother and father at the offspring age 16

Model 3c: Model 2 + subject’s BMI, age, pubertal stage and smoking, and season of the year at study

*P* values are for mean difference between risk factor groups and controls: * < 0.05. Categorical covariates are dummy-coded, with a separate dummy variable for missing values

### Maternal gestational diabetes

**Fig. 3:**
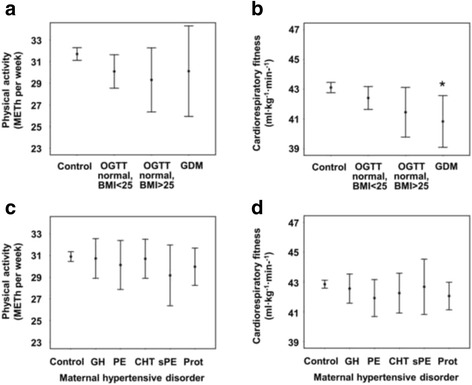
Maternal gestational diabetes (GDM) and hypertension during pregnancy predicting physical activity and cardiorespiratory fitness. Sex adjusted mean levels (95% CI) of physical activity (METh/week) and cardiorespiratory fitness (ml·kg^−1^·min^−1^) in adolescents according to exposure. Statistically significant mean differences compared with the reference category (Ref.) are shown as *p* value * < 0.05. GH = gestational hypertension; PE = preeclampsia; CHT = chronic hypertension; sPE = superimposed preeclampsia; Prot = proteinuria

Table 5, panel a (Fig. 3) shows physical activity and fitness among adolescents exposed to GDM or its risk factors than among those not exposed. Cardiorespiratory fitness was lower among adolescents whose mothers had GDM during pregnancy than among controls (Table 5, panel b).

**Table 5:** Maternal gestational diabetes (GDM) and its risk factors predicting physical activity and fitness among adolescents. Mean differences (95% CI) of physical activity (METh/week) and cardiorespiratory fitness (ml·kg^−1^·min^−1^) in adolescents exposed to maternal GDM or risk factors compared with offspring of mothers with no risk factors for GDM

	Model	No risk factors for GDM	Maternal risk factors for GDM but normal OGTT		GDM
		BMI < 25	BMI < 25	BMI > 25	Both BMI groups together
Physical activity (METh/week)		*N* = 3144	*N* = 457	*N* = 135	*N* = 74
	1	Mean 31.7 (SD 17.0)	−1.6 (−3.3;0.0)	−2.4 (−5.3;0.5)	−1.6 (−5.5;2.3)
	2		−1.8 (−3.4;-0.1)*	−1.8 (−4.7;1.1)	−1.4 (−5.3;2.5)
	3a		−1.6 (−3.3;0.1)	−1.4 (−4.4;1.6)	−0.9(−4.8;3.0)
	3b		−1.8 (−3.5;-0.2)*	−1.6 (−4.5;1.3)	−1.1 (−4.9;2.8)
	3c		−1.6 (−3.3;0.0)	−1.5 (−4.4;1.4)	−1.2 (−5.1;2.6)
Cardiorespiratory fitness (ml·kg^−1^·min^−1^)		*N* = 2528	*N* = 371	*N* = 109	*N* = 65
	1	Mean 43.2 (SD 11.4)	−0.7 (−1.7;0.3)	−1.7 (−3.3;0.0)	−2.3 (−4.4;-0.1)*
	2		−0.7 (−1.7;0.2)	−1.5 (−3.2;0.2)	−2.2 (−4.4;0.0)*
	3a		−0.6 (−1.5;0.4)	−1.3 (−3.0;0.5)	−1.7 (−3.9;0.5)
	3b		−0.7 (−1.7;0.2)	−1.5 (−3.2;0.2)	−2.1 (−4.3;0.0)
	3c		−0.5 (−1.4;0.4)	0.1 (−1.5;1.8)	−1.1 (−3.1;1.0)

Adolescents of mothers with type 1 diabetes (*n* = 15 for physical activity and 12 for cardiorespiratory fitness) were excluded from the analysis

Multiple linear regression models are adjusted for:

Model 1: sex

Model 2: sex, parental education

Model 3a: Model 2 + length of gestation, maternal hypertension, BMI of the father at the beginning of pregnancy, smoking of the mother during pregnancy

Model 3b: Model 2 + physical activity of mother and father at the offspring age 16

Model 3c: Model 2 + subject’s BMI, age, pubertal stage and smoking, and season of the year at study

*P* values are for mean difference between risk factor groups and controls: * < 0.05. Categorical covariates are dummy-coded, with a separate dummy variable for missing values

### Maternal hypertensive disorders in pregnancy

Adolescents exposed to maternal hypertensive disorders during pregnancy had similar physical activity and cardiorespiratory fitness levels as controls (Additional file 7, Fig. 3).

### Maternal smoking during pregnancy

Adolescents whose mothers smoked during pregnancy had lower cardiorespiratory fitness than those whose mothers did not (Additional file 8). Physical activity levels were similar between groups (Additional file 8).

### Adjustment for covariates

Associations of most exposures with physical activity remained similar when adjusted for parental education (Model 2, Tables 1, 2, 3, 4 and 5, Additional files 6, 7 and 8). Only the association of maternal obesity with physical activity of the offspring slightly attenuated, also remaining statistically significant (Model 2, Table 3). When the results of any exposure were further adjusted for pregnancy-related factors and father’s BMI (Model 3a), or alternatively, for parents’ physical activity level (Model 3b), there was no remarkable change in the results (Tables 1, 2, 3, 4 and 5, Additional files 6, 7 and 8).

The associations with cardiorespiratory fitness were not affected by adjustment for parental educational attainment (Model 2 in Tables 1, 2, 3, 4 and 5, Additional files 6, 7 and 8). However, further adjustments for pregnancy-related factors and father’s BMI (Model 3a) or parents’ physical activity (Model 3b) attenuated the associations with maternal BMI and risk factors for gestational diabetes to non-significance (Tables 3 and 5), while the other associations with other exposures remained similar.

When the adolescents’ own characteristics at 16 years were taken into account, the associations between prenatal determinants and physical activity remained similar, but those with cardiorespiratory fitness attenuated to non-significance (Model 3c in Tables 1, 2, 3, 4 and 5, Additional files 6, 7 and 8). This attenuation was principally due to adjusting for the adolescent’s BMI (not shown).

### Sensitivity analyses

Adolescents with asthma had a 1.2 (95% CI -0.2 to 2.6) METh/week higher level of physical activity (*n* = 608) and 0.2 (−0.7 to 1.0) ml·kg^−1^·min^−1^ lower cardiorespiratory fitness (*n* = 407). When they were excluded, all associations with physical activity remained; for cardiorespiratory fitness, associations with length of gestation the mean difference of −0.2(−0.3;-0.0) ml·kg^−1^·min^−1^ attenuated to −0.1 (−0.2;0.1) ml·kg^−1^·min^−1^ and maternal smoking the mean difference of −0.7 (−1.3;-0.1) attenuated to 0.4 (−1.0;0.2) ml·kg^−1^·min^−1^.

We further reanalyzed the data including only participants with no missing covariate data (physical activity: *n* = 3037 [45.5%]; cardiorespiratory fitness: *n* = 2405 [51.2%] of all participants). For both physical activity and cardiorespiratory fitness, the associations with birth weight, birth weight SD score, and length of gestation were strengthened, and the associations with maternal overweight and obesity and paternal overweight, as well as most associations with GDM and its risk factors, attenuated to non-significance. Other associations remained.

## Discussion

Our aim was to systematically assess prenatal and parental characteristics that underlie the association between birth weight and physical activity and cardiorespiratory fitness in adolescence. Most prenatal and parental predictors of decreased cardiorespiratory fitness were largely similar to those of decreased levels of physical activity. These included high birth weight and birth weight SD score as well as maternal and paternal overweight or obesity. Moreover, maternal GDM and smoking during pregnancy, were associated with cardiorespiratory fitness but not with physical activity in adolescence.

### Birth weight and length of gestation

Earlier findings in relation to self-reported physical activity [23–25] are illustrated by a meta-analysis of 13 population-based studies of adolescents or adults. This meta-analysis showed an inverse U-shaped association: there was no association within the normal range of birth weight, but low and high extremes were associated with lower levels of physical activity [22]. Low self-reported physical activity is also seen in studies focusing on the low extreme, those born preterm at very or extremely low birth weight [23–25], although these associations have not been captured by studies using objective measurement by accelerometry [44, 45]. Only sparse data on high birth weight and physical activity has been reported [31, 32]. Among middle-aged British adults those with a birth weight over 4.5 kg were less likely to undertake regular physical activity than individuals with birth weight between 4 and 4.5 kg, [22, 32] A combined analysis of objectively measured physical activity did not reveal an association with birth weight. However, in one of the participant cohorts comprising 12–14-year old Brazilian children higher birth weight was associated with slightly decreased total physical activity and increased sedentary time, but the difference attenuated after adjustment for gestational age [46]. Our results suggested lower physical activity levels at the higher end of the birth weight range. The association we observed was modest: one kg higher birth weight corresponded to 0.6 METh/week lower physical activity, the equivalent of 12 min less brisk walking per week. Such small differences may capture more substantial effects of conditions underlying birth weight [22]. The association we found was not explained by factors underlying high birth weight that we could assess, including maternal GDM and parental BMI. However, these factors obviously capture only a part of the variation in intrauterine environment, and it is possible that other underlying conditions such as subthreshold hyperglycemia could contribute to the association between high birth weight and lower physical activity.

We were unable to specifically replicate the associations between shorter gestation or preterm birth and physical activity. The association could be masked by the comparatively low participation rate among those born early preterm (Additional file 4) [47, 48]. However, the inverse U-shaped association between the length of gestation and physical activity is, in general, consistent with the earlier observation of lower physical activity levels among adults born very preterm [23, 24].

Studies that have assessed the association of birth weight or length of gestation with cardiorespiratory fitness show conflicting results. Two studies have used the same method, submaximal step test. Lower birth weight SD score was associated with lower cardiorespiratory fitness among 4304 31-year-olds of the NFBC 1966 [28] but not in another study of 692 23-year-olds [49]. Lower birth weight has been associated with worse cardiorespiratory fitness, also among 12-year-olds in a maximal shuttle run test [26]. This was not seen in a re-examination at 15 years [26] or in other studies among 13- to 18-year-olds undergoing a shuttle run test [30] or among 60-year-olds performing a 2 km walking test [29].

Also lower physical fitness is a general feature of those born preterm at extremely low birth weight [25, 27]. However, a recent study including all degrees of preterm birth, found no difference in cardiorespiratory fitness, although muscular fitness was lower in those born before 34 weeks of gestation [49]. In the light of this literature, our finding that those born with a high birth weight have lower cardiorespiratory fitness, which is due to both longer gestation and a more rapid fetal growth, seems counterintuitive. It could in part reflect an effect of undiagnosed gestational diabetes, as discussed in the next section, or subthreshold hyperglycaemia. It is also in line with previous [22, 32, 46] and our present results of physical activity.

The associations with longer lengths of gestation are novel. They parallel recent suggestions of increased levels of cardiometabolic risk factors among pre-pubertal children born post-term [50], and risk of obesity among adolescent males but not females [51] and also greater BMI and increased risk of overweight and obesity in a cohort comprising young women at 26 years of age [52]. A difficulty in studies on long lengths of gestation is that they strongly depend on obstetric practice. The likelihood of fetal distress increases substantially after 41 completed weeks, and current guidelines usually recommend frequent follow-up after that, with induction of delivery if signs of fetal distress are present [53, 54]. Thus long-term consequences an advanced length of gestation can in theory be a consequences of underlying causes, consequences of timely induction of delivery, or consequences of fetal distress not adequately prevented by obstetric management. Differentiating between these is potentially important but difficult in observational studies.

### Maternal pregnancy conditions and parental BMI

We found that offspring whose parents had higher prepregnancy BMI undertook less physical activity and had lower cardiorespiratory fitness. The association was similar for maternal and paternal prepregnancy BMI. Maternal GDM predicted lower cardiorespiratory fitness but not physical activity. The associations with fitness attenuated when the offspring’s characteristics at 16 years of age, including BMI, were taken into account. This suggests that the associations with cardiorespiratory fitness may be in part explained by shared genetic or environmental/lifestyle factors within the family. Also in a previous study, high maternal pre-pregnancy BMI was associated with lower offspring cardiorespiratory fitness which was mediated via offspring BMI [55].

It is also of note that in 1985–1986 GDM screening was performed only for high-risk mothers, 13% of those in the source cohort [38]. Consequently, many GDM cases are likely to have gone undiagnosed resulting in small numbers. This results in reduced power, although intrauterine exposure to maternal GDM in this cohort does predict an adverse metabolic phenotype at 16 years of age [38].

We found that adolescents exposed to maternal smoking during pregnancy had lower cardiorespiratory fitness, although the difference was small. Previous study on NFBC 1986 involving a sub-group re-examined at 19 years also suggests a lower cardiorespiratory fitness in 1–2 min running test among individuals exposed to maternal smoking [55]. Maternal smoking during pregnancy is a well-known risk factor for both small birth size and preterm birth [56], as well as later adverse health outcomes [19–21]. Our results together with the previous finding suggests that a part of this association may be mediated through lower cardiorespiratory fitness, although the association may also be confounded by unmeasured lifestyle factors [57, 58].

### Public health implications

Although the associations we found are relatively modest, they have public health implications. They argue that the benefits prevention and optimal treatment of conditions such as maternal overweight/obesity and GDM are likely to extend to offspring long-term health. They also reinforce previous suggestions that prenatal risk factors can be used to identify at-risk children early. Many of the risk factors relate to shared family lifestyle which can be targeted in prenatal and child healthcare.

### Study strengths and limitations

The strengths of this study include physical activity and cardiorespiratory fitness assessed in the same individuals, the high participation rate, and a large unselected birth cohort with reliable data on birth weight, verified length of gestation, and the diagnoses of maternal GDM and hypertensive disorders.

However, follow-up data on both physical activity and cardiorespiratory fitness would be required to evaluate possible causal relationships. Also, an objective measurement of physical activity would improve the accuracy of quantification of physical activity, and maximal exercise testing with the direct measurement of oxygen uptake would provide a more precise estimate of cardiorespiratory fitness. The sex-adjusted correlation coefficient 0.22 between physical activity and cardiorespiratory fitness is fairly low but consistent with other studies assessing the relationship of physical activity and cardiorespiratory fitness in adolescence [59, 60]. Physical activity at school was not assessed in the present study and it is likely that not all types of physical activity were captured by this questionnaire. These factors leading to underestimation of performed physical activity may reduce correlation together with other confounding.

A lower participation rate of some exposure groups, such as those born early preterm or with a low socio-economic status, could cause selection bias (Additional files 4 and 5). Earlier studies show that a low socioeconomic status predicts lower levels of both leisure time physical activity and cardiorespiratory fitness [47, 48], as well as preterm birth [61]. Such selection bias would be expected to dilute rather than exaggerate the observed differences between groups and may be one reason why we could not replicate previous findings of low physical activity and cardiorespiratory fitness in adolescents born preterm.

## Conclusions

High birth weight and underlying prenatal conditions, including maternal and paternal obesity, are associated with both lower levels of physical activity and cardiorespiratory fitness in adolescence. In addition, longer length of gestation and maternal GDM predict lower cardiorespiratory fitness. These findings suggest that the prevention and optimal treatment of pregnancy conditions potentially extend their benefits long ahead to the health of the offspring. Many of the risk factors are likely to relate to shared family lifestyle which can be targeted in prenatal and child healthcare.

## Additional files

Additional file 1: Flow chart of the NFBC 1986 study population. (DOC 32 kb)

Additional file 2: Mean (SD) values of physical activity (METh per week) and cardiorespiratory fitness (ml·kg^−1^·min^−1^) according to related perinatal factors. (DOC 104 kb)

Additional file 3: Questions used in the assessment of physical activity of the 16-year old participants of the Northern Finland Birth Cohort 1986. (DOC 31 kb)

Additional file 4: Characteristics of the participants and non-participants of the physical activity study. (DOC 88 kb)

Additional file 5: Characteristics of the participants and non-participants of the cardiorespiratory fitness study. (DOC 81 kb)

Additional file 6: Covariates in association with physical activity and fitness among adolescents. Mean differences (95% CI) of physical activity (METh per week) and cardiorespiratory fitness (ml·kg^−1^·min^−1^) compared with the reference group. (DOC 97 kb)

Additional file 7: Mean differences (95% CI) of physical activity (METh per week) and cardiorespiratory fitness (ml·kg^−1^·min^−1^) in adolescents exposed to maternal hypertensive disorders during pregnancy compared with controls. (DOC 48 kb)

Additional file 8: Mean differences (95% CI) of physical activity (METh/week) and cardiorespiratory fitness (ml·kg^−1^·min^−1^) in adolescents exposed to maternal smoking during pregnancy compared with offspring of non-smoking mothers. (DOC 37 kb)

## Abbreviations

BMI: Body mass index
CHT: Chronic hypertension
CPA: Commuting physical activity
GDM: Gestational diabetes mellitus
GH: Gestational hypertension
LPA: Light physical activity
MET: Metabolic equivalent
MVPA: Moderate-to-vigorous physical activity
NFBC: Northern Finland birth cohort
OGTT: Oral glucose tolerance test
PE: Preeclampsia
Prot: Proteinuria
SD: Standard deviation
sPE: Superimposed preeclampsia
