# Supplementary material for: Prenatal determinants of physical activity and cardiorespiratory fitness in adolescence – Northern Finland Birth Cohort 1986 study
Source: BMC Public Health. 2017 Apr 20;17:346. doi: 10.1186/s12889-017-4237-4 (PMC5399469; doi:10.1186/s12889-017-4237-4)
Supplement: Supplementary file 1 — Flow chart of the NFBC 1986 study population. (DOC 32 kb) [file 12889_2017_4237_MOESM1_ESM.doc]

Additional file 1. Figure. Flow chart of the NFBC 1986 study population

Liveborn

N = 9,432

Original cohort

N = 9,479

Invited to the study at 16 yrs N = 9,215

Stillborn

N = 47

Responded to

the postal questionnaire

N = 7,344

Attended

the clinical examination

N = 6,798

Exclusions:

No appropriate data on physical activity N = 425

Twin/triple N = 161

Mental disability N = 50

Cerebral palsy N = 7

Congenital heart defect N = 6

Blindness N = 2

Other condition restricting physical ability N = 11

Physical activity,

self-reported

N = 6,682

Exclusions:

No appropriate data on submaximal bicycle ergometry test N = 1,934

Twin/triple N = 121

Mental disability N = 24

Cerebral palsy N = 6

Congenital heart defect N = 2

Blindness N = 1

Other condition restricting physical ability N = 4

Cardiorespiratory fitness, submaximal bicycle ergometry N = 4,706
