# Supplementary material for: Prenatal determinants of physical activity and cardiorespiratory fitness in adolescence – Northern Finland Birth Cohort 1986 study
Source: BMC Public Health. 2017 Apr 20;17:346. doi: 10.1186/s12889-017-4237-4 (PMC5399469; doi:10.1186/s12889-017-4237-4)
Supplement: Supplementary file 2 — Mean (SD) values of physical activity (METh per week) and cardiorespiratory fitness (ml·kg−1·min−1) according to related perinatal factors. (DOC 104 kb) [file 12889_2017_4237_MOESM2_ESM.doc]

Additional file 2. Table. Mean (SD) values of physical activity (METh per week) and cardiorespiratory fitness (ml·kg^-1^·min^-1^) according to related perinatal factors.

|  |  | | **Physical activity (METh per week)** | | **Cardiorespiratory fitness**  **(ml·kg^-1^·min^-1^)** | |
| --- | --- | --- | --- | --- | --- | --- |
|  |  | | **N** | **Mean (SD)** | **N** | **Mean (SD)** |
|  |  | |  |  |  |  |
| **Birth weight (g)** | **< 1,500** | | 15 | 22.2 (15.5) | 11 | 39.1 (11.6) |
|  | **> 1,500 to 2,000** | | 33 | 32.5 (17.1) | 28 | 43.1 (13.9) |
|  | **> 2,000 to 2,500** | | 113 | 31.6 (15.6) | 82 | 42.2 (9.2) |
|  | **> 2,500 to 3,000** | | 567 | 30.8 (16.3) | 353 | 40.9 (9.6) |
|  | **> 3,000 to 3,500** | | 2,149 | 30.6 (16.9) | 1,483 | 42.6 (11.1) |
|  | **> 3,500 to 4,000** | | 2,536 | 30.8 (16.9) | 1,825 | 42.9 (10.8) |
|  | **> 4,000 to 4,500** | | 1,052 | 30.9 (16.9) | 780 | 43.0 (10.3) |
|  | **> 4,500** | | 217 | 29.5 (16.0) | 144 | 43.7 (9.5) |
|  |  | |  |  |  |  |
| **Birth weight SD score** | **< -2** | | 119 | 30.2 (15.9) | 79 | 42.2 (11.2) |
|  | **-2 to 1** | | 745 | 31.0 (16.9) | 504 | 43.0 (9.7) |
|  | **-1 to 1** | | 4,670 | 30.8 (16.9) | 3,324 | 42.9 (11.0) |
|  | **1 to 2** | | 943 | 30.5 (16.8) | 670 | 42.0 (10.2) |
|  | **> 2** | | 198 | 28.73 (14.87) | 124 | 40.1 (9.0) |
|  |  | |  |  |  |  |
| **Length of gestation (weeks + days)** | **< 33 + 6** | | 56 | 27.2 (16.5) | 37 | 42.0 (12.3) |
|  | **34 + 0–36 + 6** | | 197 | 31.8 (16.7) | 144 | 42.9 (9.4) |
|  | **37 + 0–42 + 0** | | 6,170 | 30.8 (16.8) | 4,335 | 42.7 (10.8) |
|  | **> 42 + 1** | | 252 | 29.3 (16.7) | 185 | 41.7 (9.2) |
|  |  | |  |  |  |  |
| **Maternal BMI before pregnancy, kg/m2** | **< 20** | | 1,594 | 30.7 (16.8) | 1,122 | 43.7 (11.1) |
|  | **20 to 25** | | 3,881 | 31.0 (16.9) | 2,760 | 42.6 (10.7) |
|  | **25 to 30** | | 829 | 30.5 (16.5) | 576 | 41.7 (10.4) |
|  | **> 30** | | 229 | 27.4 (16.7) | 148 | 39.8 (9.9) |
|  |  | |  |  |  |  |
| **Paternal BMI at the beginning of pregnancy, kg/m2** | **< 20** | | 235 | 29.6 (16.2) | 165 | 44.1 (16.6) |
|  | **20 to 25** | | 3,600 | 31.1 (16.8) | 2,517 | 42.8 (10.7) |
|  | **25 to 30** | | 1,619 | 30.0 (16.6) | 1,141 | 42.0 (9.9) |
|  | **> 30** | | 163 | 28.9 (16.4) | 108 | 41.3 (9.0) |
|  |  | |  |  |  |  |
| **Maternal risk factors for GDM** | **No risk factors for GDM** | | 3,144 | 31.7 (17.0) | 2,528 | 43.2 (11.4) |
|  | **Risk factors but normal OGTT** | **BMI < 25** | 457 | 30.3 (16.7) | 371 | 42.9 (9.7) |
|  |  | **BMI > 25** | 135 | 29.5 (17.4) | 109 | 42.7 (10.7) |
|  | **GDM** | | 74 | 30.5 (18.2) | 65 | 42.4 (9.0) |
|  |  | |  |  |  |  |
| **Maternal hypertensive disorder during pregnancy** | **Normotensive** | | 5,234 | 30.9 (16.9) | 3,678 | 42.9 (10.7) |
|  | **Gestational hypertension** | | 327 | 30.8 (16.9) | 247 | 42.8 (10.4) |
|  | **Preeclampsia** | | 201 | 30.2 (16.4) | 147 | 42.0 (9.5) |
|  | **Chronic hypertension** | | 322 | 30.6 (16.4) | 232 | 42.0 (12.6) |
|  | **Superimposed preeclampsia** | | 114 | 28.9 (15.3) | 72 | 41.6 (9.4) |
|  | **Proteinuria** | | 368 | 29.8 (16.9) | 248 | 41.6 (10.4) |
|  |  | |  |  |  |  |
| **Any maternal hypertensive disorder during pregnancy** | **Normotensive** | | 5,234 | 30.9 (16.9) | 3,678 | 42.9 (10.7) |
|  | **Gestational or chronic hypertension or preeclampsia (including superimposed preeclampsia)** | | 964 | 30.4 (16.4) | 698 | 42.2 (10.9) |
|  |  | |  |  |  |  |
| **Maternal smoking during pregnancy** | **Non-smoker** | | 5,373 | 30.8 (16.6) | 3,819 | 42.7 (10.5) |
|  | **Smoker** | | 1,309 | 30.3 (17.5) | 887 | 42.5 (11.7) |
|  |  | |  |  |  |  |
