# Supplementary material for: Prenatal determinants of physical activity and cardiorespiratory fitness in adolescence – Northern Finland Birth Cohort 1986 study
Source: BMC Public Health. 2017 Apr 20;17:346. doi: 10.1186/s12889-017-4237-4 (PMC5399469; doi:10.1186/s12889-017-4237-4)
Supplement: Supplementary file 3 — Questions used in the assessment of physical activity of the 16-year old participants of the Northern Finland Birth Cohort 1986. (DOC 31 kb) [file 12889_2017_4237_MOESM3_ESM.doc]

**Additional file 3. Questions used in the assessment of physical activity of the 16-year old participants of the Northern Finland Birth Cohort 1986**

**In Finnish:**

**46. Kuinka kauan kävelet, pyöräilet tai kuljet muilla liikuntaa vaativilla tavoilla koulumatkoilla päivittäin?** (yhteensä meno ja tulomatka)

- en lainkaan
- alle 20 minuuttia päivässä
- 20-39 minuuttia päivässä
- 40-59 minuuttia päivässä
- vähintään tunnin päivässä

**48. Kuinka paljon yhteensä harrastat ripeää liikuntaa kouluajan ulkopuolella?** (hengästyt ja hikoilet ainakin lievästi)

- en lainkaan
- noin ½ tuntia viikossa
- noin tunnin viikossa
- 2-3 tuntia viikossa
- noin 4-6 tuntia viikossa
- 7 tuntia tai enemmän viikossa

**49. Kuinka paljon yhteensä harrastat edellisen lisäksi kevyttä liikuntaa kouluajan ulkopuolella?** (et hengästy ja hikoile)

- en lainkaan
- noin ½ tuntia viikossa
- noin tunnin viikossa
- 2-3 tuntia viikossa
- noin 4-6 tuntia viikossa
- 7 tuntia tai enemmän viikossa

**In English:**

**46. How many minutes altogether does it take you to walk, bike or otherwise physically move to get to school and home from school daily?**

- Not at all
- Less than 20 minutes a day
- 20-39 minutes a day
- 40-59 minutes a day
- More than an hour a day

**48. Outside the school hours, how many hours a week altogether do you participate in brisk physical activity?** (causing at least some sweating and getting out of breath)

- Not at all
- About ½ hour a week
- About an hour a week
- 2-3 hours a week
- About 4-6 hours a week
- 7 hours a week or more

**49. In addition to physical activity described above, how many hours a week altogether do you spend in light physical activity?** (causing no sweating and getting out of breath)

- Not at all
- About ½ hour a week
- About an hour a week
- 2-3 hours a week
- About 4-6 hours a week
- 7 hours a week or more
