# Supplementary material for: Prenatal determinants of physical activity and cardiorespiratory fitness in adolescence – Northern Finland Birth Cohort 1986 study
Source: BMC Public Health. 2017 Apr 20;17:346. doi: 10.1186/s12889-017-4237-4 (PMC5399469; doi:10.1186/s12889-017-4237-4)
Supplement: Supplementary file 4 — Characteristics of the participants and non-participants of the physical activity study. (DOC 88 kb) [file 12889_2017_4237_MOESM4_ESM.doc]

Additional file 4. Table. Characteristics of the participants and non-participants of the physical activity study.

|  | **Physical activity analysis**  **N = 6,682** | **n** | **n missing** | **All non-participants of physical activity analysis N = 2,539** | **N** | **N missing** | **p value**^1^ |
| --- | --- | --- | --- | --- | --- | --- | --- |
| **Boys, N (%)** | 3,189 (47.7) | 6,682 | 0 | 1,567 (61.7) | 2,538 | 1 | < 0.0001 |
| **Multiple birth, N (%)** | 0 | 6,682 |  | 223 (8.7) | 2,539 |  | < 0.0001 |
| **Mother smoked during pregnancy, N (%)** | 1,309 (19.6) | 6,682 |  | 699 (27.5) | 2,539 |  | < 0.0001 |
| **Maternal BMI before pregnancy, kg/m2 (SD)** | 22.28 (3.36) | 6,533 | 129 | 22.50 (3.75) | 2,456 |  | 0.009 |
| **< 20, N (%)** | 1,594 (24.4) |  |  | 611 (24.9) |  |  |  |
| **20 to 25, N (%)** | 3,881 (59.4) |  |  | 1,376 (56.0) |  |  |  |
| **25 to 30, N (%)** | 829 (12.7) |  |  | 357 (14.5) |  |  |  |
| **> 30, N (%)** | 229 (3.5) |  |  | 112 (4.6) |  |  |  |
| **Paternal BMI before pregnancy, kg/m2 (SD)** | 24.03 (2.71) | 5,617 | 1,065 | 24.12(2.71) | 1,968 |  | 0.216 |
| **< 20, N (%)** | 235 (4.2) |  |  | 73 (3.7) |  |  |  |
| **20 to 25, N (%)** | 3,600 (64.1) |  |  | 1,265 (64.3) |  |  |  |
| **25 to 30, N (%)** | 1,619 (28.8) |  |  | 577 (29.3) |  |  |  |
| **> 30, N (%)** | 163 (2.9) |  |  | 53 (2.7) |  |  |  |
| **Prenatal/neonatal** |  |  |  |  |  |  |  |
| **Birth weight, g (SD)** | 3,587 (514) | 6,682 | 0 | 3,424 (696) | 2,539 | 0 | < 0.0001 |
| **Birth weight SD score (SD)** | 0.07 (0.99) | 6,675 | 7 | -0.14 (1.16) | 2,533 | 6 | < 0.0001 |
| **< -2, N (%)** | 119 (1.8) |  |  | 115 (4.5) |  |  |  |
| **-2 to 1, N (%)** | 745 (11.2) |  |  | 408 (16.1) |  |  |  |
| **-1 to 1, N (%)** | 4,670 (70.0) |  |  | 1,627 (64.2) |  |  |  |
| **1 to 2, N (%)** | 943 (14.1) |  |  | 318 (12.6) |  |  |  |
| **> 2, N (%)** | 198 (3.0) |  |  | 65 (2.6) |  |  |  |
| **Gestational age, weeks (SD)** | 39.90 (1.53) | 6,675 | 7 | 39.30 (2.43) | 2,534 | 5 | < 0.0001 |
| **< 33+6, N (%)** | 56 (0.8) |  |  | 108 (4.3) |  |  |  |
| **34 + 0-36 + 6, N (%)** | 197 (3.0) |  |  | 136 (5.4) |  |  |  |
| **37 + 0-42 + 0, N (%)** | 6,170 (92.4) |  |  | 2,185 (86.2) |  |  |  |
| **> 42 + 1, N (%)** | 252 (3.8) |  |  | 105 (4.1) |  |  |  |
| **Risk factors for maternal gestational diabetes, N** |  | 4,931 | 1,751 |  | 495 | 2,044 | 0.395 |
| **Gestational diabetes, N (%)** | 75 (1.5) |  |  | 10 (2.0) |  |  |  |
| **OGTT ind not perf, N (%)** | 1,103 (22.4) |  |  | 111 (22.4) |  |  |  |
| **OGTT normal, N (%)** | 609 (12.4) |  |  | 72 (14.5) |  |  |  |
| **OGTT not ind, N (%)** | 3,144 (63.8) |  |  | 302 (61.0) |  |  |  |
| **Maternal hypertensive disorders, N** |  | 6,566 | 116 |  | 2,237 | 302 | 0.940 |
| **Gestational hypertension, N (%)** | 327 (5.0) |  |  | 120 (5.4) |  |  |  |
| **Preeclampsia, N (%)** | 201 (3.1) |  |  | 74 (3.3) |  |  |  |
| **Chronic hypertension, N (%)** | 322 (4.9) |  |  | 109 (4.9) |  |  |  |
| **Superimposed, N (%)** | 114 (1.7) |  |  | 39 (1.7) |  |  |  |
| **Proteinuria, N (%)** | 368 (5.6) |  |  | 117 (5.2) |  |  |  |
| **Normotensive, N (%)** | 5,234 (79.7) |  |  | 1,778 (79.5) |  |  |  |

^1^Differences between participants and non-participants were evaluated with χ^2^-tests for categorical variables and Student’s t-test for continuous variables.
