# Supplementary material for: Prenatal determinants of physical activity and cardiorespiratory fitness in adolescence – Northern Finland Birth Cohort 1986 study
Source: BMC Public Health. 2017 Apr 20;17:346. doi: 10.1186/s12889-017-4237-4 (PMC5399469; doi:10.1186/s12889-017-4237-4)
Supplement: Supplementary file 5 — Characteristics of the participants and non-participants of the cardiorespiratory fitness study. (DOC 81 kb) [file 12889_2017_4237_MOESM5_ESM.doc]

**Additional file 5. Table. Characteristics of the participants and non-participants of the cardiorespiratory fitness study.**

|  | **Cardiorespiratory fitness N = 4,706** | **N** | **N missing** | **All non-participants of cardiorespiratory fitness analysis N = 4,515** | **N** | **N missing** | **p value^1^** |
| --- | --- | --- | --- | --- | --- | --- | --- |
| **Boys, N(%)** | 2,513(53.4) | 4,706 | 0 | 2,243 (49.7) | 4,514 | 1 | < 0.0001 |
| **Multiple birth, N (%)** | 0 | 4,706 | 0 | 223 (4.9) | 4,515 | 0 | < 0.0001 |
| **Mother smoked during pregnancy, N (%)** | 887 (18.8) | 4,706 |  | 1,121 (24.8) | 4,515 |  | < 0.0001 |
| **Maternal BMI before pregnancy, kg/m2 (SD)** | 22.24 (3.30) | 4,606 | 100 | 22.45 (3.64) | 4,383 | 132 | < 0.003 |
| **< 20, N (%)** | 1,122 (24.4) |  |  | 1,083 (24.7) |  |  |  |
| **20 to 25, N (%)** | 2,760 (60.0) |  |  | 2,497 (57.0) |  |  |  |
| **25 to 30, N (%)** | 576 (48.6) |  |  | 610 (13.9) |  |  |  |
| **> 30, N (%)** | 148 (3.2) |  |  | 193 (4.4) |  |  |  |
| **Paternal BMI before pregnancy, kg/m2 (SD)** | 24.04 (2.68) | 3,931 | 775 | 24.07 (2.75) | 3,654 | 861 | 0.627 |
| **< 20** | 165 (4.2) |  |  | 143 (3.9) |  |  |  |
| **20 to 25** | 2,517 (64.0) |  |  | 2,348 (64.3) |  |  |  |
| **25 to 30** | 1,141 (29.0) |  |  | 1,055 (28.9) |  |  |  |
| **> 30** | 108 (2.7) |  |  | 108 (3.0) |  |  |  |
| **Prenatal/neonatal** |  |  |  |  |  |  |  |
| **Birth weight, g (SD)** | 3,600 (0.51) | 4,706 | 0 | 3.842 (630) | 4,515 | 0 | < 0.0001 |
| **Birth weight SD score (SD)** | 0.08 (0.98) | 4,701 | 5 | -0.06 (1.15) | 4,507 | 8 | < 0.0001 |
| **< -2** | 79 (1.7) |  |  | 155 (3.4) |  |  |  |
| **-2 to 1** | 504 (10.7) |  |  | 649 (14.4) |  |  |  |
| **-1 to 1** | 3,324 (70.7) |  |  | 2,973 (66.0) |  |  |  |
| **1 to 2** | 670 (14.3) |  |  | 591 (13.1) |  |  |  |
| **> 2** | 124 (2.6) |  |  | 139 (3.1) |  |  |  |
| **Gestational age, weeks (SD)** | 39.90 (1.52) | 4,701 | 5 | 39.56 (2.10) | 4,508 | 7 | < 0.0001 |
| **< 33 + 6, N (%)** | 37 (0.8) |  |  | 127 (2.8) |  |  |  |
| **34 + 0-36 + 6, N (%)** | 144 (3.1) |  |  | 189 (4.2) |  |  |  |
| **37 + 0-42 + 0, N (%)** | 4,335 (92.1) |  |  | 4,020 (89.2) |  |  |  |
| **> 42 + 1, N (%)** | 185 (3.9) |  |  | 172 (3.8) |  |  |  |
| **Risk factors for maternal gestational diabetes, N** |  | 3,954 | 752 |  | 1,472 | 3,043 | 0.510 |
| **Gestational diabetes** | 65 (1.6) |  |  | 20 (1.4) |  |  |  |
| **OGTT ind not perf** | 867 (21.9) |  |  | 347 (23.6) |  |  |  |
| **OGTT normal** | 494 (12.5) |  |  | 187 (12.7) |  |  |  |
| **OGTT not ind** | 2,528 (63.9) |  |  | 918 (62.4) |  |  |  |
| **Maternal hypertensive disorders, N** |  | 4,624 | 82 |  | 4,179 | 336 | 0.556 |
| **Gestational hypertension, N (%)** | 247 (5.3) |  |  | 200 (4.8) |  |  |  |
| **Preeclampsia, N (%)** | 147 (3.2) |  |  | 128 (3.1) |  |  |  |
| **Chronic hypertension, N (%)** | 232 (5.0) |  |  | 199 (4.8) |  |  |  |
| **Superimposed, N (%)** | 72 (1.6) |  |  | 81 (1.9) |  |  |  |
| **Proteinuria, N (%)** | 248 (5.4) |  |  | 237 (5.7) |  |  |  |
| **Normotensive, N (%)** | 3,678 (79.5) |  |  | 3,334 (79.8) |  |  |  |

^1^Differences between participants and non-participants were evaluated with χ^2^-tests for categorical variables and Student’s t-test for continuous variables.
