# Supplementary material for: Prenatal determinants of physical activity and cardiorespiratory fitness in adolescence – Northern Finland Birth Cohort 1986 study
Source: BMC Public Health. 2017 Apr 20;17:346. doi: 10.1186/s12889-017-4237-4 (PMC5399469; doi:10.1186/s12889-017-4237-4)
Supplement: Supplementary file 6 — Covariates in association with physical activity and fitness among adolescents. Mean differences (95% CI) of physical activity (METh per week) and cardiorespiratory fitness (ml·kg−1·min−1) compared with the reference group. (DOC 97 kb) [file 12889_2017_4237_MOESM6_ESM.doc]

**Additional file 6. Table. Covariates in association with physical activity and fitness among adolescents. Mean differences (95% CI) of physical activity (METh per week) and cardiorespiratory fitness (ml·kg^-1^·min^-1^) adjusted for sex compared with the reference group.**

|  |  | **Physical activity (METh per week)** | | **Cardiorespiratory fitness**  **(ml·kg^-1^·min^-1^)** | |
| --- | --- | --- | --- | --- | --- |
|  |  | **N** | **Mean difference (95% CI)**^1^ | **N** | **Mean difference (95% CI)**^1^ |
|  |  |  |  |  |  |
| **Parental educational level** | **Elementary** | 305 | -4.7 (-6.8;-2.5) | 180 | -2.3 (-3.7;-1.0) |
|  | **High school** | 2,134 | -5.0 (-6.3;-3.7) | 1,428 | -1.0 (-1.8;-0.3) |
|  | **Intermediate** | 2,531 | -1.9 (-3.2;-3.7) | 1,794 | -0.7 (-1.4;0.1) |
|  | **University** | 907 | Referent | 656 | Referent |
|  | **Missing** | 805 | -4.3 (-5.9;-2.7) | 648 | -1.2 (-2.1;-0.3) |
|  |  |  |  |  |  |
| **BMI of the subject (kg/m2)** | **< 20** | 2,831 | -2.3 (-3.2;-1.5) | 2,008 | 2.0 (1.5;2.4) |
|  | **20 to 25** | 3,071 | Referent | 2,225 | Referent |
|  | **25 to 30** | 563 | -2.2 (-3.7;-0.7) | 362 | -5.9 (-6.7;-5.0) |
|  | **> 30** | 175 | -8.4 (-10.9;-5.9) | 104 | -11.6 (-13.1;-10.0) |
|  | **Missing** | 42 | -1.6 (-6.6;3.5) | 7 | -3.0 (-8.8;2.8) |
|  |  |  |  |  |  |
| **Age at study (years)** |  | 6,682 | -2.6 (-3.6;-1.6) | 4,706 | -0.3 (-0.9;0.4) |
|  |  |  |  |  |  |
| **Current smoking of the subject** |  | 6,682 | -2.5(-3.4;-1.6) | 4,706 | -0.1 (-0.7;0.5) |
|  |  |  |  |  |  |
| **Tanner stage, organs** | **I, II, III** | 1,593 | -2.5 (-3.7;-1.3) | 1,253 | 0.1 (-0.5;0.7) |
|  | **IV** | 2,957 | -2.1 (-3.2;-1.1) | 2,395 | 0.0 (-0.6;0.6) |
|  | **V** | 1,302 | Referent | 1,031 | Referent |
|  | **Missing** | 830 | -3.7 (-5.1;-2.2) | 27 | 1.1 (-2.0;4.3) |
|  |  |  |  |  |  |
| **Tanner stage, pubic hair** | **I, II, III** | 1,452 | 0.0 (-1.3;1.3) | 1,078 | 0.6 (-0.2;1.3) |
|  | **IV** | 2,732 | 0.3 (-0.8;1.3) | 2,164 | 0.8 (0.2;1.4) |
|  | **V** | 1,676 | Referent | 1,442 | Referent |
|  | **Missing** | 822 | -1.8 (-3.2;-0.4) | 22 | -0.3 (-3.7;3.2) |
|  |  |  |  |  |  |
| **Season of the year** | **Spring** | 468 | -2.5 (-4.1;-0.9) | 1,130 | 0.1 (-0.8;1.1) |
|  | **Summer** | 4,870 | Referent | 382 | Referent |
|  | **Autumn** | 848 | -2.6 (-3.8;-1.4) | 1,865 | 0.3 (-0.6;1.3) |
|  | **Winter** | 291 | -4.2 (-6.1;-2.2) | 1,325 | 0.17 (-0.8;1.1) |
|  | **Missing** | 205 | -1.4 (-3.8;0.9) | 4 | 4.9 (-3.2;13.1) |
|  |  |  |  |  |  |
| **Physical activity of mother (frequency)** | **once a month** | 984 | -3.1 (-4.4;-1.8) | 643 | -1.0 (-1.7;-0.2) |
|  | **2–3 times per month** | 682 | -0.9 (-2.4;0.5) | 449 | -0.6 (-1.5;0.3) |
|  | **once a week** | 1,396 | -1.4 (-2.5; -0.3) | 936 | -1.0 (-1.7;-0.3) |
|  | **2–3 times per week** | 1,921 | Referent | 1,373 | Referent |
|  | **4–5 times per week** | 687 | 1.5 (0.0;2.9) | 484 | 0.3 (-0.6;1.1) |
|  | **Missing** | 1,048 | -2.4 (-3.7;-1.2) | 821 | -1.0 (-1.7;-0.3) |
|  |  |  |  |  |  |
| **Physical activity of father (frequency)** | **once a month** | 1,158 | -4.2 (-5.4; -2.9) | 775 | -0.9 (-1.6;-0.1) |
|  | **2–3 times per month** | 689 | -0.4 (-1.9; 1.1) | 487 | -0.5 (-1.4;0.4) |
|  | **once a week** | 1,203 | -0.9 (-2.2; 0.3) | 827 | -0.3 (-1.1;0.4) |
|  | **2–3 times per week** | 1,564 | Referent | 1,114 | Referent |
|  | **4–5 times per week** | 613 | 0.9 (-0.6;2.5) | 442 | 0.5 (-0.4;1.4) |
|  | **Missing** | 1,455 | -2.7 (-3.9;-1.5) | 1,063 | -0.8 (-1.5; -0.1) |
