# Supplementary material for: Prenatal determinants of physical activity and cardiorespiratory fitness in adolescence – Northern Finland Birth Cohort 1986 study
Source: BMC Public Health. 2017 Apr 20;17:346. doi: 10.1186/s12889-017-4237-4 (PMC5399469; doi:10.1186/s12889-017-4237-4)
Supplement: Supplementary file 7 — Mean differences (95% CI) of physical activity (METh per week) and cardiorespiratory fitness (ml·kg−1·min−1) in adolescents exposed to maternal hypertensive disorders during pregnancy compared with controls. (DOC 48 kb) [file 12889_2017_4237_MOESM7_ESM.doc]

Additional file 7. Table. Mean differences (95% CI) of physical activity (METh per week) and cardiorespiratory fitness (ml·kg^-1^·min^-1^) in adolescents exposed to maternal hypertensive disorder during pregnancy compared with controls^1^.

|  | **Model** | **Offspring of normotensive mothers** | **Maternal hypertensive disorder during pregnancy** | | | | |
| --- | --- | --- | --- | --- | --- | --- | --- |
|  |  |  | **Gestational hypertension** | **Preeclampsia** | **Chronic hypertension** | **Superimposed PE** | **Proteinuria** |
| **Physical activity (METh per week)** |  | N=5,234 | N=327 | N=201 | N=322 | N=114 | N=368 |
|  | **1** | Mean  30.9 (SD 16.9) | -0.2 (-2.0;1.7) | -0.8 (-3.1;1.6) | -0.2 (-2.1;1.7) | -1.7 (-4.8;1.4) | -0.9 (-2.7;0.8) |
|  | **2** |  | 0.0 (-1.9;1.9) | -0.8 (-3.1;1.5) | 0.0 (-1.9;1.9) | -1.4 (-4.4;1.7) | -0.8 (-2.6;0.9) |
|  | **3a** |  | 0.1 (-1.8;2.0) | -0.5 (-2.9;1.8) | 0.4 (-1.5;2.2) | -0.8 (-3.9;2.3) | -0.7 (-2.4;1.1) |
|  | **3b** |  | -0.1 (-1.9;1.7) | -0.5 (-2.9;1.8) | -0.2 (-2.0;1.7) | -1.8 (-4.9;1.3) | -0.7 (-2.5;1.0) |
|  | **3c** |  | -0.2 (-2.0;1.7) | -1.1 (-3.4;1.2) | 0.1 (-1.7;2.0) | -1.0 (-4.1;2.1) | -0.9 (-2.6;0.8) |
|  |  |  |  |  |  |  |  |
| **Cardiorespiratory fitness (ml·kg^-1^·min^-1^)** |  | N=3678 | N=247 | N=147 | N=232 | N=72 | N=248 |
|  | **1** | Mean  42.9 (SD 10.7) | -0.3 (-1.4;0.8) | -0.9 (-2.2;0.4) | -0.6 (-1.7;0.5) | -0.2 (-2.1;1.8) | -0.8 (-1.9;0.3) |
|  | **2** |  | -0.3 (-1.3;0.8) | -0.9 (-2.3;0.4) | -0.5 (-1.6;0.6) | -0.1 (-2.1;1.8) | -0.8 (-1.8;0.3) |
|  | **3a** |  | -0.1 (-1.1;1.0) | -0.7 (-2.1;0.7) | -0.1 (-1.2;1.0) | 0.6 (-1.4;2.5) | -0.5 (-1.6;0.5) |
|  | **3b** |  | -0.3 (-1.3;0.8) | -0.9 (-2.2;0.5) | -0.6 (-1.7;0.5) | -0.2 (-2.1;1.8) | -0.8 (-1.9;0.3) |
|  | **3c** |  | 0.2 (-1.9;0.7) | -0.6 (-1.9;0.7) | -0.2 (-1.2;0.9) | 0.7 (-1.1;2.5) | -0.4 (-1.4;0.6) |

^1^Control group consists of adolescents whose mothers were normotensive during pregnancy.

Multiple linear regression models:

Model 1: sex

Model 2: sex, parental education

Model 3: sex, parental education, length of gestation, maternal GDM, maternal hypertension, maternal BMI before pregnancy, paternal BMI before pregnancy, maternal smoking

Model 4: Model 3 + BMI, age, pubertal stage, smoking, season
