# Supplementary material for: Prenatal determinants of physical activity and cardiorespiratory fitness in adolescence – Northern Finland Birth Cohort 1986 study
Source: BMC Public Health. 2017 Apr 20;17:346. doi: 10.1186/s12889-017-4237-4 (PMC5399469; doi:10.1186/s12889-017-4237-4)
Supplement: Supplementary file 8 — Mean differences (95% CI) of physical activity (METh/week) and cardiorespiratory fitness (ml·kg−1·min−1) in adolescents exposed to maternal smoking during pregnancy compared with offspring of non-smoking mothers. (DOC 37 kb) [file 12889_2017_4237_MOESM8_ESM.doc]

**Additional file 8. Table. Mean differences (95% CI) of physical activity (METh/week) and cardiorespiratory fitness (ml·kg^-1^·min^-1^**) in adolescents exposed to maternal smoking during pregnancy compared with offspring of non-smoking mothers.

|  | **Model** | **Offspring of non-smoking mothers** | **Maternal smoking during pregnancy** |
| --- | --- | --- | --- |
| **Physical activity (METh/week)** |  | N = 5,373 | N = 1,309 |
| **N = 6,682** | **1** | Mean 30.82 (SD 16.62) | -0.5 (-1.5;0.6) |
|  | **2** |  | 0.3 (-0.7;1.3) |
|  | **3a** |  | 0.3 (-0.7;1.4) |
|  | **3b** |  | 0.6 (-0.4;1.6) |
|  | **3c** |  | 0.7 (-0.3;1.8) |
| **Cardiorespiratory fitness**  **(ml·kg^‑1^·min^‑1^)** |  | N = 3,819 | N = 887 |
| **N = 4,706** | **1** | Mean 42.74 (SD 10.48) | -0.7 (-1.3;-0.1)* |
|  | **2** |  | -0.5 (-1.1;0.1) |
|  | **3a** |  | -0.6 (-1.2;0.0)* |
|  | **3b** |  | -0.5(-1.1;0.1) |
|  | **3c** |  | 0.1(-0.5;0.7) |

Multiple linear regression models are adjusted for:

Model 1: sex

Model 2: sex, parental education

Model 3a: Model 2 + length of gestation, maternal GDM, maternal hypertension, BMI of the mother before pregnancy, BMI of the father at the beginning of pregnancy, smoking of the mother during pregnancy

Model 3b: Model 2 + physical activity of mother and father at study

Model 3c: Model 2 + subject’s BMI, age, pubertal stage and smoking, and season of the year at study

P values are for mean difference between risk factor groups and controls: * < 0.05. Categorical covariates are dummy-coded, with a separate dummy variable for missing values.
